# Treatment of Elderly Patients with Colorectal Cancer

**DOI:** 10.1155/2018/2176056

**Published:** 2018-03-11

**Authors:** Yoshiro Itatani, Kenji Kawada, Yoshiharu Sakai

**Affiliations:** Department of Surgery, Graduate School of Medicine, Kyoto University, Kyoto 606-8507, Japan

## Abstract

Colorectal cancer (CRC) is one of the leading causes of cancer-related deaths worldwide. As society ages, the number of elderly patients with CRC will increase. The percentage of patients with right-sided colon cancer and the incidence of microsatellite instability are higher in elderly than in younger patients with CRC. Moreover, the higher incidence of comorbid diseases in elderly patients indicates the need for less invasive treatment strategies. For example, care should be taken in performing additional surgery after endoscopic submucosal dissection for elderly patients with high-risk T1 CRC. Minimally invasive surgery, such as laparoscopic colectomy, would be preferable for elderly patients with CRC. Chemotherapy for elderly patients requires careful monitoring for adverse events. The aim of this review is to summarize the clinicopathological features of CRC in elderly patients, optical surgical strategies, including endoscopic and laparoscopic resection, and chemotherapeutic strategies, including postoperative adjuvant chemotherapy and systemic chemotherapy for unresectable CRC.

## 1. Introduction

Expansion of the worldwide population and elevation of life expectancy have increased the number of elderly individuals, resulting in aging of the population. According to the United Nations Population Fund (UNFPA), life expectancy around the world elevated from 64.8 years to 70 years over the past 20 years. Moreover, by 2050, people aged ≥60 years will account for almost 22% of the world's population, reaching over 2 billion people [1]. Because the incidence of many cancers is higher in patients aged ≥65 years, the number of elderly patients with cancers is expected to increase markedly.

Colorectal cancer (CRC) is one of the most common causes of cancer deaths worldwide [2–4], and the global incidence of CRC continues to increase [5]. In clinical practice, increased numbers of elderly patients with CRC undergo surgery and/or receive chemotherapy. These individuals are more likely than young patients to have comorbidities, such as cardiovascular disease, respiratory disease, renal dysfunction, and/or liver dysfunction, making treatment riskier. Physical activity is usually evaluated by measuring the performance status (PS) scoring of Eastern Cooperative Oncology Group (ECOG), but it is sometimes difficult to determine [6]. Furthermore, aging itself can reduce physiological recuperative power. In fact, aging is an independent risk factor for both in-hospital morbidity and mortality after colorectal surgery [7–9]. Therefore, designing appropriate treatment strategies for elderly patients with CRC requires comprehensive understanding of the characteristics of CRC in these patients. Here, we would like to review clinicopathological features and molecular alterations of CRC in elderly patients, as well as the optimal surgical approaches (i.e., endoscopic resection and laparoscopic surgery), and chemotherapy.

## 2. Clinicopathological Features and Genetic Background of CRC in Elderly Patients

### 2.1. Clinical Characteristics

**Figure 1:**
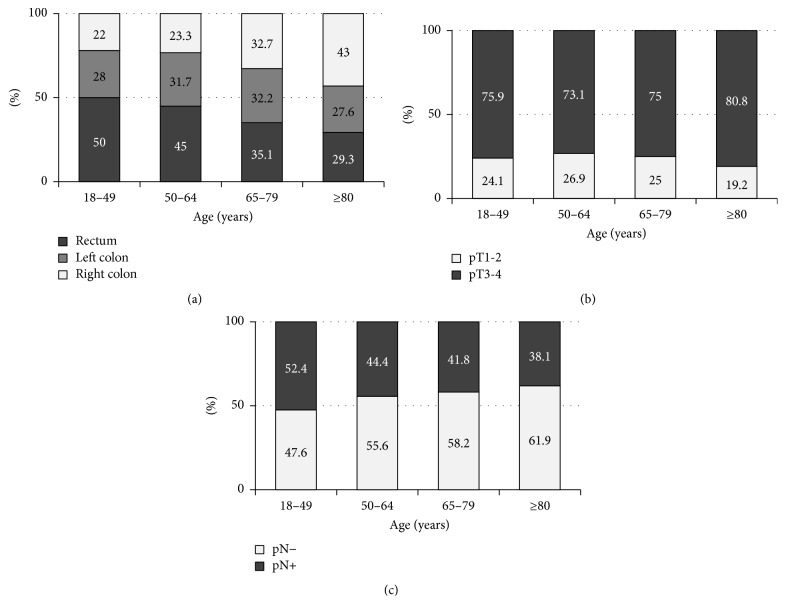
(a) Proportion of tumor location at indicated ages [10]. (b) Proportion of pathological T factor at indicated ages [10]. (c) Proportion of lymph node metastases at indicated ages [10].

**Figure 2:**
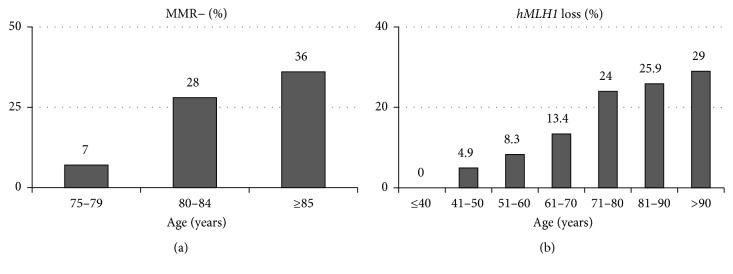
(a) Proportion of MMR-deficient CRC at indicated ages [12]. (b) Proportion of* hMLH1* loss in CRC at indicated ages [13].

One of the most prominent clinical characteristics of elderly, compared to younger, patients with CRC is their higher frequency of right-sided colon cancer. This incidence increases with patient age, reaching about 50% in patients with CRC aged ≥80 years (Figure 1(a)) [10, 11]. Moreover, the incidence of right-sided colon cancer is about 10% higher in women than in men aged ≥80 years [11]. Although elderly patients tend to have large and locally invasive CRC, the frequency of lymph nodes metastasis is lower compared to that in younger ones (Figures 1(b) and 1(c)) [10]. Mismatch repair- (MMR-) deficient cancer with microsatellite instability (MSI) is more frequent in elderly patients with CRC, being present in 36% of patients aged ≥85 years, with an especially high frequency in women (Figure 2(a)) [12, 13]. In these right-sided and MSI-high CRC developed in the elderly women,* hMLH1* gene promoter is frequently methylated and its protein expression is silenced (Figure 2(b)) [13–15].

In a mouse model of MSI colonic adenoma that carries* loxP* sites flanking exon 14 of* Adenomatous polyposis coli (Apc)* gene regulated by* CDX2* promoter and a long mononucleotide tract* (CDX2P9.5-G22Cre;Apc*^flox/flox^), most of the adenomatous lesions can be developed in the proximal colon [16]. These data are consistent with the fact that the incidence of MSI-high CRC is higher in right-sided colon than in left-sided colon or rectum in elderly patients [17, 18].

### 2.2. Pathological Characteristics and Genetic Background

**Figure 3:**
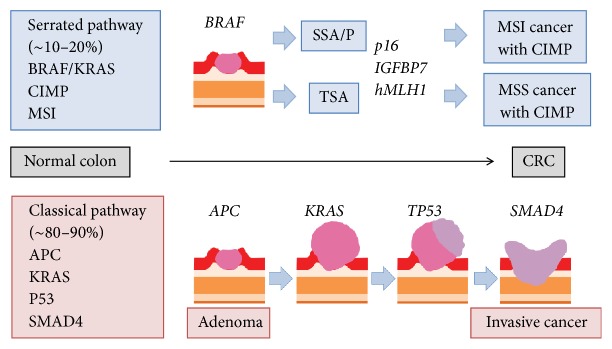
Schematic representation of the hypothesis of CRC carcinogenesis. Upper section shows serrated pathway, and lower one shows classical pathway.

Mucinous carcinoma and serrated adenocarcinoma are often found in elderly patients [19, 20]. The serrated pathway is one of the evolutionary steps of CRC carcinogenesis (Figure 3) [21]. The serrated pathway starts from the progression of serrated polyps, including traditional serrated adenomas (TSAs) and sessile serrated adenomas/polyp (SSA/P) [22]. TSA tends to develop in the left-sided colon and rectum, whereas SSA/P tends to develop in the right-sided colon. TSAs can have two types of molecular characteristics: one having* KRAS* mutations and the other having* BRAF* mutations [22]. CRCs that develop from TSAs seldom exhibit MSI, but develop into microsatellite stable (MSS) tumors. In contrast, SSA/Ps can give rise to CRCs with high MSI [23]. Most SSA/Ps are primed by* BRAF* mutations, followed by bearing CpG island methylator phenotype (CIMP), and it finally becomes MSI-high CRC [22–24]. Although CIMP is a genome-wide phenotype, methylation of the* p16*,* insulin growth factor binding protein 7 (IGFBP7)*, and* hMLH1* is important for the development of MSI-high CRC [24]. Acquiring the MSI phenotype is the key step of malignant progression from SSA/P, as this phenotype increases the likelihood of mutations in the microsatellite genomic region, resulting in an invasive phenotype.

### 2.3. Medullary Adenocarcinoma

Medullary adenocarcinoma is a rare pathological type of CRC. This type of poorly differentiated adenocarcinoma has a phenotype indicative of minimal or no glandular differentiation [25]. The clinicopathological characteristics of medullary adenocarcinoma include its predominant location in the right-sided colon, its higher incidence in elderly women, and its relatively better prognosis despite its poorly differentiated phenotype. MSI is high in these tumors, along with* hMLH1* promoter methylation [25]. Histologically, medullary carcinomas consist of a small uniform population of tumor cells with prominent nucleoli and eosinophilic cytoplasm. These cells grow in a solid-sheet structure, often containing Crohn's-like lymphoid reaction (CLR) and intratumoral lymphocytic infiltration [26]. CLR represents peritumoral lymphoid aggregates located couple of millimeters beyond the advancing tumor fronts [27]. Tumor-infiltrating lymphocytes (TILs) consist of T-cell population, and are frequently found in CRCs with high MSI [28]. The presence of CLR and TILs reflects strong antitumor immunity, and it is a good prognostic indicator for CRC after adjustment of traditional staging [29]. These data are consistent with the recent finding that immune checkpoint inhibitors are effective in the patients with MSI-high CRC [30].

## 3. Surgical Approaches for Elderly Patients with CRC

### 3.1. Endoscopic Resection

Endoscopic resection is a minimally invasive approach for adenomas and early cancers. Endoscopic submucosal dissection (ESD) is ideal because of its en bloc resection. Although less invasive than surgery, ESD still carries risks of perforation (6%) and bleeding (1%) [43]. Therefore, caution should be exercised in performing ESD for elderly patients, as they are more likely to have comorbidities that can exacerbate post-ESD complications. However, aging itself is not a contraindication for ESD, as it has been shown to be effective and safe for elderly patients with CRC, with en block resection rates of 81.2–96.3%, perforation rates of 1.8–6.1%, and bleeding rates of 3.0–3.7% [44–46]. Moreover, the 5-year disease specific survival (DFS) rates in the elderly populations have been reported to be almost 100% when appropriately managed [45].

Some patients with early CRC who undergo endoscopic resection require additional colectomy with lymph node dissection, because about 10% of patients with T1 CRC have lymph node metastases [47]. Indications for additional surgery in patients with T1 tumors include (1) depth of submucosal invasion ≥ 1000 *μ*m, (2) vascular invasion (i.e., lymphatic or venous invasion) positive, (3) poorly differentiated adenocarcinoma, signet-ring cell carcinoma, or mucinous carcinoma, or (4) grade 2/3 budding at the site of deepest invasion [47, 48]. To date, there is no consensus regarding whether additional surgery is really effective and reasonable for elderly patients who have T1 CRC with such signs as described above.

### 3.2. Laparoscopic Surgery for Elderly Patients with CRC

Aging is an independent risk factor in major digestive surgery [49]. Laparoscopic surgery for CRC was widely adopted in the late 1990s to 2000s because it was regarded as minimally invasive. However, at first, application for laparoscopic surgery was limited and sometimes elderly and/or high-risk patients were excluded just because this surgery required techniques different from those of open surgery and standardized procedure had not been established. Recent randomized controlled trials have reported that laparoscopic surgery for CRC has an equivalent oncological result and better short-term outcomes compared with open surgery [50–56]. Moreover, analyses of large databases have found that laparoscopic surgery is an independent predictor of reduced mortality after CRC surgery [57–61]. Taken together, these studies emphasize the benefits of laparoscopic colectomy over open surgery, including reduced invasiveness, lower mortality rates, shorter hospital stay, and lower costs, with comparable oncological outcomes.

Minimally invasive laparoscopic colectomy is therefore indicated for elderly patients with CRC. Some observational studies have shown that laparoscopic surgery has better short-term outcomes than open surgery for elderly patients with CRC (Table 1) [31–35, 62, 63]. Most of these studies have reported that postoperative hospital stay is shorter after laparoscopic than after open surgery, suggesting that laparoscopic surgery may reduce surgical complications. Similar to nonelderly patients, elderly patients should undergo curative laparoscopic colectomy with D3 lymph node dissection, when an operation under the general anesthesia is possible due to a lack of severe comorbidities. Moreover, care should be taken, especially in elderly patients, to maintain postoperative activities of daily living (ADL) and quality of life (QOL). In particular, the anus-sparing surgery for low rectal cancer (i.e., low anterior resection or intersphincteric resection) can cause the postoperative defecation dysfunction, and so we need to determine the operative method considering the preoperative anal function.

## 4. Chemotherapy for Elderly Patients with CRC

### 4.1. General Management of Chemotherapy

Particular attention is required when planning chemotherapy for elderly cancer patients, because of reductions in organ function and preexisting comorbidities. The kidneys and livers are the most important organs involved in the pharmacokinetics and pharmacodynamics of chemotherapy agents. For example, doses of capecitabine and TS-1, both of which are frequently used to treat CRC, should be reduced or omitted in patients with renal dysfunction [64, 65], and doses of irinotecan should be reduced in patients with hepatic dysfunction [66]. Bevacizumab, an antivascular endothelial growth factor (VEGF) neutralizing antibody, sometimes causes proteinuria in a dose-dependent manner, requiring its reduction or discontinuation [67].

### 4.2. Adjuvant Chemotherapy (Table 2)

Unfortunately, some patients experience recurrence/metastasis even after complete resection of the primary CRC. The likelihood of recurrence after curative resection may be reduced by administration of adjuvant chemotherapy. A randomized trial sponsored by the National Cancer Institute reported, in 1990, that adjuvant therapy with fluorouracil plus levamisole (5-FU/LEV) reduced recurrence risk by 41% over surgery alone for patients with stage III (metastatic lymph node-positive) CRC [68]. Based on the results of this trial and other following trials, a regimen of 5-FU plus folic acid (leucovorin) (5-FU/LV) became a standard treatment for stage III CRC [37–39]. Many randomized controlled trials have tested other adjuvant chemotherapy regimens. For example, the addition of oxaliplatin to 5-FU/LV resulted in acceptable tolerance and a better DFS and/or overall survival (OS) rate in patients with stage III CRC patients (MOSAIC trial [40, 42] and NSABP C-07 [41]). Results of the MOSAIC trial, however, found that elderly patients aged ≥65 years did not benefit from adding oxaliplatin to 5-FU/LV, suggesting the need for care in applying the results of these randomized trials to adjuvant chemotherapy for elderly patients with CRC.

Most of these randomized trials did not include many elderly patients. For example, the two major trials described above included only 25 (1%) and 131 (5%) patients aged ≥75 years, respectively [69]. A large cohort study, published in 2002, of elderly patients aged ≥67 years with stage III CRC reported a survival benefit of 5-FU-based adjuvant chemotherapy over surgery alone [70]. Another cohort study published in 2006 also reported its benefit in patients aged ≥65 years [71]. However, the age predilection of CRC suggests that patients in their late 60s are not “elderly.” In 2012, Sanoff et al. reported a cohort study combining four large databases of patients diagnosed as stage III CRC between 2004 and 2007. A total of 5,489 patients with stage III CRC aged ≥75 years were analyzed using covariate adjusted and propensity score-matched proportional hazards models. Compared with surgery alone, 5-FU-based adjuvant chemotherapy had significant survival benefit, whereas the addition of oxaliplatin to 5-FU-based chemotherapy provided no significant benefit over 5-FU alone, although it tended to improve prognosis [69].

### 4.3. Chemotherapy for Unresectable CRC

Because of the discovery of novel drugs, including molecular targeting reagents, systemic chemotherapy for advanced/metastatic CRC dramatically has increased median overall survival by 2-3 years these days. However, most of these clinical trials did not include patients with CRC aged ≥75 years, because these trials were usually designed for the patients without any comorbidities. It is uncertain, therefore, whether the results of these clinical trials can be applicable to elderly patients with CRC.

A pooled analysis of the safety and efficacy of oxaliplatin in elderly patients with CRC was reported in 2006 [72]. Although this analysis mixed trials of the FOLFOX4 regimen as adjuvant, first-line, and second-line settings, it included 614 patients with CRC aged ≥70 years. That analysis found that the incidence of grade ≥3 hematologic toxicities (neutropenia and thrombocytopenia) was significantly higher in elderly than in other patients. In contrast, the incidence of other adverse events, such as neurotoxicity, infection, diarrhea, nausea/vomiting, and fatigue, and the overall incidence of grade ≥3 toxicity were not associated with older age. Moreover, the benefits of FOLFOX over control treatment, as determined by response rate, progression-free survival (PFS), DFS, and OS, were not associated with age, suggesting that oxaliplatin-containing chemotherapy is efficient and safe for the elderly patients with CRC.

The randomized trial (MRC FOCUS2) was designed for the elderly and frail CRC patients who needed the reduced dosage of chemotherapy regimen [73]. In this trial, 42% (191/459) were aged ≥76 years, and the starting doses were 80% of the standard doses, with discretionary escalation to full dose after 6 weeks. They identified that adding oxaliplatin onto a 5-FU-based regimen exhibited some improvement of PFS, although not statistically significant.

## 5. Conclusion

Aging is one of the factors we need to take into account in determining a comprehensive strategy of CRC treatment. Several studies reported that aging itself was an independent prognostic factor in these patients. To date, there is not enough evidence to develop a standardized treatment of elderly patients with CRC. A personalized strategy is required, considering each patient's comorbidities, performance status, and life styles.

## Figures and Tables

**Table 1:** Representative studies comparing laparoscopic colectomy and open surgery for elderly CRC patients.

Author (year)	Age	Hospital stay (days)^*∗*^	*P*	OS^*∗∗*^	*P*
Cummings (2012) [31]	≥65	8.3 ± 6.2 versus 10 ± 8.9	<0.001	55.8% versus 50.05% (5 y)	0.095
Mukai (2014) [32]	≥85	14.7 versus 21.7	<0.0001	–	–
Vallribera Valls (2014) [33]	75–84	10 versus 14.3	0.001	–	–
	≥85	11.4 versus 15.4	0.077	–	–
Nakamura (2014) [34]	≥85	10 versus 19	<0.0001	–	–
Hinoi (2015) [35]	≥80	12 versus 13.0 (colon)	<0.001	85.5% versus 81.2% (colon, 3 y)	0.916
		19 versus 18 (rectum)	0.990	78.6% versus 70.2% (rectum, 3 y)	0.765

^*∗*^Laparoscopic surgery versus open surgery; ^*∗∗*^percentage of survival at indicated years in parentheses. y, years; –, not mentioned in the article.

**Table 2:** Representative studies of adjuvant chemotherapy for stage II and/or stage III CRC.

Author (year)	Regimen	DFS^*∗∗*^	*P*	OS^*∗∗*^	*P*
Moertel (1995) [36]	5-FU/LEV versus none	63% versus 47% (3.5 y)	<0.0001	71% versus 55% (3.5 y)	0.0064
Francini (1994) [37]	5-FU/LV versus none	74% versus 59% (5 y)	0.005	79% versus 65% (5 y)	0.0044
IMPACT (1995) [38]	5-FU/LV versus none	71% versus 62% (3 y)	<0.0001	83% versus 78% (3 y)	0.018
O'Connell (1997) [39]	5-FU/LV versus none	74% versus 58% (5 y)	0.001	74% versus 63% (5 y)	0.01
André (2004) [40]	FL + Oxali versus FL	78% versus 73% (3 y)	0.002	–	–
Kuebler (2007) [41]	FLOX versus FULV	73% versus 67% (4 y)	0.0034	–	–
André (2009) [42]	FOLFOX4 versus LV5FU2	66% versus 59% (5 y)	0.005	73% versus 69% (6 y)	0.023

^*∗∗*^Percentage of survival at indicated years in parentheses. y, years; –, not mentioned in the article.

## Abbreviations

Apc: Adenomatous polyposis coli
ADL: Activity of daily living
CIMP: CpG island methylator phenotype
CLR: Crohn's-like lymphoid reaction
CRC: Colorectal cancer
DFS: Disease-free survival
ECOG: Eastern Cooperative Oncology Group
ESD: Endoscopic submucosal dissection
IGFBP: Insulin growth factor binding protein
MMR: Mismatch repair
MSI: Microsatellite instability/instable
MSS: Microsatellite stable
OS: Overall survival
PFS: Progression-free survival
PS: Performance status
QOL: Quality of life
SSA/P: Sessile serrated adenoma/polyp
TIL: Tumor-infiltrating lymphocyte
TSA: Traditional serrated adenomas
VEGF: Vascular endothelial growth factor.
